# Developing a policy game intervention to enhance collaboration in public health policymaking in three European countries

**DOI:** 10.1186/s12889-017-4963-7

**Published:** 2017-12-19

**Authors:** H. P. E. M. Spitters, J. A. M. van Oers, P. Sandu, C. J Lau, M. Quanjel, D. Dulf, R. Chereches, L. A. M. van de Goor

**Affiliations:** 1 Department of Tranzo, Tilburg School of Social and Behavioral Sciences, Tilburg University, P.O. Box 90153, 5000 LE Tilburg, The Netherlands; 2 National Institute of Public Health and the Environment (RIVM), P.O. Box 1, 3720 BA Bilthoven, The Netherlands; 3 Department of Public Health, Faculty of Political, Administrative and Communication Sciences, University Babes-Bolyai, 7 Pandurilor St. Universitas, Room 910, Zip code 400376 Cluj-Napoca, Romania; 4 Research Centre for Prevention and Health, Centre of Health, The Capital Region of Denmark, Rigshospitalet – Glostrup, Ndr. Ringvej 57, Building 84/85, -2600 Glostrup, DK Denmark; 5 Entrea, Youth Care, Special Needs Education and Research, P.O. Box 6546, 6503 GA Nijmegen, The Netherlands

**Keywords:** Policy game, Public health policymaking, Stakeholder network, Cross-sector collaboration

## Abstract

**Background:**

One of the key elements to enhance the uptake of evidence in public health policies is stimulating cross-sector collaboration. An intervention stimulating collaboration is a policy game. The aim of this study was to describe the design and methods of the development process of the policy game ‘In2Action’ within a real-life setting of public health policymaking networks in the Netherlands, Denmark and Romania.

**Methods:**

The development of the policy game intervention consisted of three phases, pre intervention, designing the game intervention and tailoring the intervention.

**Results:**

In2Action was developed as a role-play game of one day, with main focus to develop in collaboration a cross-sector implementation plan based on the approved strategic local public health policy.

**Conclusions:**

This study introduced an innovative intervention for public health policymaking. It described the design and development of the generic frame of the In2Action game focusing on enhancing collaboration in local public health policymaking networks. By keeping the game generic, it became suitable for each of the three country cases with only minor changes. The generic frame of the game is expected to be generalizable for other European countries to stimulate interaction and collaboration in the policy process.

## Background

*General background* Policymaking is a dynamic process, dependent on competing sources of input such as the political agenda, ideas and interests, timing, different sources of evidence and the involved stakeholder network [1–5]. This dynamic process is also applicable to public health policymaking. Furthermore, public health policies are very complex, because of the many interconnected problems and determinants outside the health sector [6, 7]. Hence, it is advocated to take into account aspects like involvement of stakeholders, governance structures including committees and working groups [8], and different sources of knowledge, using a cross-sector approach in the policymaking process to facilitate the development of integrated public health policies. It is advocated that these type of policies are more effective [9, 10], especially when these are supported by the best available evidence [2, 3].

However, the uptake of different sources of knowledge in policy, i.e. scientific evidence, stakeholder expertise and other knowledge is not straightforward [11–13] and depends on the topic of the policy [14, 15]. A recent review by Oliver et al., looking into the barriers and facilitators of the use of evidence by policymakers shows that key facilitators for the uptake of knowledge are relationships and collaboration in stakeholder networks [16]. Also other studies advocate for a close interaction and collaboration between policy, practice and research communities, with the purpose to increase the dissemination and uptake of different kinds of knowledge [11, 17–19]. In this line of thoughts, Cairney provides key strategies to stimulate the uptake of evidence in policymaking. The strategies are i) building on networks which entail both researchers and policy makers, ii) working on collaboration, profitable for the whole network and iii) working on good relationships. Yet, this study also emphasizes the potential shortcomings of these strategies [20].

Exploration of the potential of strengthening relations, interactions and collaboration within stakeholder networks in the policymaking process is needed.

Understanding the relations between stakeholders in the network is one thing [21], intervening in an effective way in the stakeholder network, to stimulate the interaction, communication and collaboration, is another thing. A possible intervention which takes the network into account and aims at stimulating collaboration between stakeholders in the network can be a policy game. Policy games can be described as tailored interventions meant to initiate change and are commonly used methods in organizations that are preparing for change [22–24]. Mayer defined policy games as: *“experi(m)ent(i)al, rule-based, interactive environments, where players learn by taking actions and by experiencing their effects through feedback mechanisms that are deliberately built into and around the game”* [25]. Effective elements of policy games are the (direct link to and) use of a simplified version of reality, while keeping main stakeholders, their relations and the dynamicity unchanged [26, 27]. Games intend to give participants a way to reflect on using their own abilities to deal with complex collaborative projects. Therefore, games until now were mainly used as a learning tool to practice communication and collaboration and increase the understanding of group processes [22]. This was done in different settings and various research domains, such as organization science, operations research, management science, but also health care and education [22, 24, 28–30]. Evaluations of these games showed a positive influence on collaboration, increased understanding of the problem and possible solutions, because of collective wisdom and insight in the role of each stakeholders’ organization [30, 31].

*Background policy games* Various terminologies are used for the policy game intervention, such as simulation games, policy exercise, policy games or serious games, with at least the commonality that real-life stakeholders participate in an artificial setting that reflects (aspects of) reality [24]. In this paper, we use the term policy game.

Policy games are interactive, participatory approaches, taking the real-life situation as a starting point [24, 29, 31]. They can be seen as a workshop where several instruments, such as brainstorm elements and workshop elements are brought together to tackle a problem [23, 24, 29, 30, 32]. The aim of policy games is to initiate change in a stakeholder network (inter-organizational or between independent organizations), by policy exploration, decision making and/or strategic change [23, 24, 30, 33]. In policy games, a theory or hypothesis is tested, by involving real-life stakeholders [23, 24, 30, 33]. The real-life stakeholders (game participants), are allocated roles and play a game under certain rules, to create a future following the steps in the game [23, 24, 29, 30, 32]. This is done by reducing the complex system situation by capturing the essential aspects in an artificial environment resembling real-life [24, 27]. In the development of the policy game it is important that the key perspectives that influence the process at stake, are represented (e.g. key stakeholders, key challenges in the network and key elements in the structure where the stakeholders are working) [23, 24, 26, 27].

Three of the working mechanisms in games, which make them a useful exercise, are the setting, the time constraint in the game, and the cycles embedded in the game.

The setting, the artificial environment as created in a game, provides a safe environment. This enables participants to go beyond their own habits and behavior of everyday life [23, 28]. Furthermore, playing roles reminds participants that it is a ‘game’ that they are ‘playing’. Their imagination and creativity are required for productive communication. These two elements together give the game participants the opportunity to explore together new behavior and strategies by creating and analyzing the future, without the possibility to fail [23, 28], but with the possibility to learn.

Second is the time constraint. A game acts as a pressure cooker where time is precious which makes participants act on their natural behavior and habits [28].

Third, the cycles provide the structure of the policy game. This structure provides a learning experience for the whole network at stake [23, 24, 28, 30, 34]. Policy games generally consist of several cycles within the game and entail learning by doing over the course of the intervention. Essential within a cycle are the decisions made by participants, its results (meeting the purpose of the game) and not least the evaluation of the process. After a cycle an evaluation takes place to reflect and discuss the results and decisions made [23, 24, 30, 35, 36]. These learning experiences are brought into a next cycle. The game is finalized by a debriefing session in which participants translate their learning experiences to their real-world setting [23, 37]. Learning experiences and insight might be expected in 5 categories, described by Duke and Geurts, i.e. complexity, communication, creativity, consensus and commitment to action [23, 24]. The stakeholders learn about the system they work in, actively experience problems together and how they could solve these problems.

In our study we aimed to develop a policy game for public health networks of three European countries. Since policy games to date are not yet applied to stakeholder networks in different countries, we focus on the detailed description of the design and development of the game intervention. Therefore the aim of this study is to describe the design and methods of the development process of a policy game intervention within public health policymaking networks, more specific in Health Enhancing Physical Activity (HEPA) policies in the Netherlands, Denmark and Romania. Our purpose was to develop a generic frame of a policy game and apply a tailored game to three European country cases, which could support collaboration in local public health policymaking networks.

## Methods

### Intervention design and settings

The policy game intervention was developed and piloted in three different country cases as part of the larger REPOPA (REsearch into POlicy to enhance Physical Activity) project. REPOPA aimed to facilitate the development of more evidence-informed policies in physical activity with the involvement of seven countries [38]. The three countries at stake were: the Netherlands, Denmark and Romania.

A policy game is a context-oriented intervention [24], generally developed for only one specific context. In this case, the policy game intervention was aimed to fit in different countries’ context. Therefore, in each country, a research team of two public health researchers was present, having specific knowledge and expertise of the HEPA policymaking process of their own country. Together with policy officers (key figures) of the case they examined and discussed how the policymaking process evolved in that specific municipality, to get a good understanding of the country case’s context. The involvement of key figures in an early stage of the development process of the game is highly recommended in the gaming literature [23, 24]. In this way a direct link was formed to each country case in the development process of the game. In the Netherlands and Denmark this key figure group consisted of persons of the local authority and other stakeholders next to the researchers (Table 1). In Romania, policy implementation is more ad hoc and therefore instead of forming a key figure group, interviews were held with individual stakeholders, to identify needs (Table 2) and whom to interview next.

**Table 1:** Context of the three country cases

Case	The Netherlands	Denmark	Romania
Size of the city	Average size municipality	Average size municipality	Highly populated municipality with a high student population
Stage policy	New developed health policy, working towards an HEPA implementation plan.	New developed health policy, and needed an implementation plan, including HEPA	HEPA policy plan was in the development phase
Focus policy	Mainly on physical activity promotion for youth	Mainly on physical activity promotion for youth and citizens with special needs and chronic diseases	The development of the local HEPA Strategy for 2014–2020, “Sport and Community”
Responsible for the local policy	- Local administrative level of the municipality for the HEPA policy; - Regional Sport Service was assigned the development and implementation of the HEPA plan	- Local administrative level in the sector Health and Care for the health policy - The implementation of the policy was a common responsibility (across sectors in the municipality)	- Centralized administrative system -National ministries responsible for the policy development - None of the local/county stakeholders take responsibility for the lead in the implementation of the HEPA policy
Entry point important for case to be represented in the game	- Representation of a specific neighborhood - Representation that makes involvement of new stakeholder possible	- Representation of the municipality - Representation of specific deprived community areas	- Representation was open, i.e. local participants from different stakeholder groups. - Representation of some members of the physical activity working group
Group of key figures	Five representatives of the Dutch case: - One policy maker of the municipality, sector health and welfare - One policy advisor of the regional public health service - One policy advisor of the regional sport service - Two researchers of the Tilburg University	Five representatives of the Danish case: - Two policy makers from the Health sector, represented by a team coordinator and an administrative civil servant, - One policy maker from the Culture and Leisure sector - Two researcher of the Research Centre for Prevention and Health	Two representatives of the Romanian case - Two researchers of the Babes-Bolyai University, Cluj-Napoca - Interviewed several individual stakeholders along the way^a^

^a^A different approach was used in Romania, because of the different knowledge base of the network

**Table 2:** Specific needs of each of the country cases

SPECIFIC NEEDS IN EACH OF THE COUNTRY CASES	
The Netherlands	- Give a boost to cross-sector implementation of the HEPA policy plan - Enhance the understanding of the policy development process - Understand the needs and values of other organizations in the stakeholder network and what each other’s gain would be to participate in the HEPA implementation plan - Make a connection of the learning experiences in the game to other health areas than physical activity - Gain more insight in new divisions of tasks and how to work with different budget allocations
Denmark	- Use the policy game as a kick-off opportunity to start the development of the implementation plan and get ideas and plans how to develop the plan - Focus on the necessity of a joint effort across municipality sectors - Identify ideas for processes that could be of use for future developed policy plans - Strengthen knowledge exchange with stakeholders and what is needed to fulfill this task - Learn to become more visible in the process
Romania	- Need to take a step back of the current developed strategy and get first a more general overview of the local needs to be able to address these local needs - To enhance own knowledge and understanding on the roles of other local stakeholders in local HEPA policymaking - Increase collaboration based on common needs and goals - Overcome barriers in the policy process, such as scarce communication between stakeholders, scarce resources, different goals among stakeholders and lack of interest from the local public authority

**Fig. 1:**
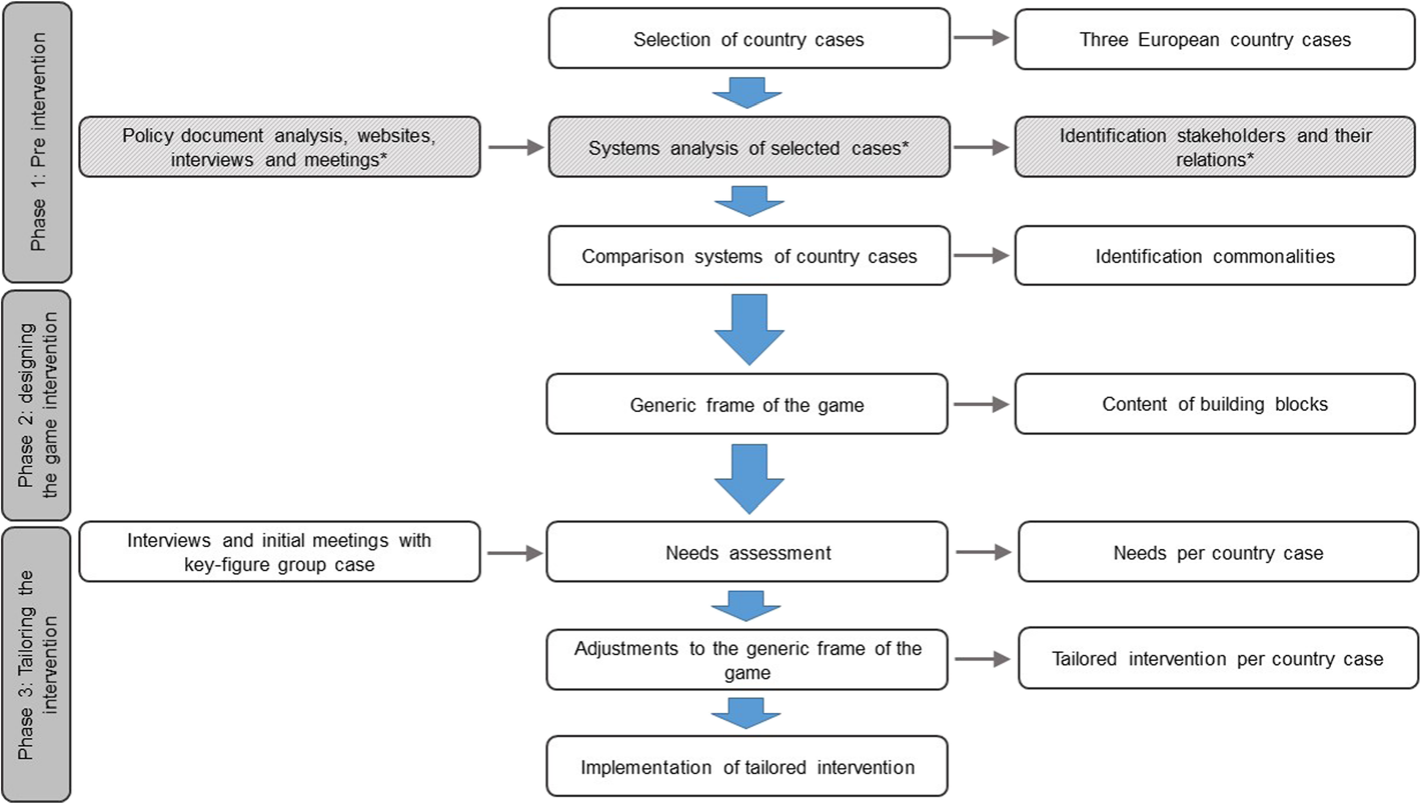
Phases of the development of the policy game intervention, implemented as part of the REPOPA-project. * Grey striped parts are described elsewhere [21]

We have gone through the three phases, described below and summarized in Fig. 1, to identify what specific subject each of the cases wanted to focus on and what their envisioned achievements were. We developed the policy game based on the existing literature [23, 24, 29, 39]. Because our aim was to develop one game useful for three different countries, the development process of this games was adjusted accordingly, e.g. by comparting the three systems and by adding the third phase ‘tailoring the intervention’ (See Fig. 1). This resulted in the following phases: phase 1 (pre intervention) covers the initial preparation; phase 2 (designing the game intervention); and phase 3 (tailoring the intervention). Together the phases cover the development of the policy game intervention. In the process of developing the intervention, a game expert was involved. The policy game intervention is called In2Action.

### Main phases of the development of the policy game intervention In2Action

Phase 1, the pre intervention phase, consisted of the selection of cases, a systems analysis of these selected cases and a comparison of systems and cases between countries [21]. The systems analyses covered the essential aspects of the system in a schematic model, which was required to support the course and interaction of participants in the policy game [23, 24].

Phase 2 covered the generic frame of the policy game, which consists of building blocks of the intervention based on identified commonalities across countries. In designing the game it was important to capture *“the integrality and creativity of the systems analysis by incorporating the best ideas into the game”* [23]. Furthermore, the format of the game was chosen. In this case a role-play game is chosen, close to the real-life situation. Based on the systems analyses, the identified problem and envisioned achievements, the game’s building blocks (role description, script, rules, events and participants) are formed, which together make the generic frame of the policy game intervention In2Action.

Phase 3 covered the tailored game, in which the generic frame of the game is adjusted to the needs and differences specific for each country case. This phase included a needs assessment of the cases, tailoring the frame of the policy game, and the implementation of the intervention. Before implementing the game in the case, it was important to test run it with informed and trusted participants, to know if all elements of the tailored game were in place [23, 24, 29, 39]. Testing the game was not only important as a test, but also as a means of validation; represented the simulated problem/environment in the game the real-life problem.

## Results

The results section follows the three phases for developing the policy game In2Action, phase 1: pre intervention; phase 2: designing the game intervention; and phase 3: tailoring the intervention.

### Phase 1: Pre intervention phase

#### Selection of country cases

In the pre intervention phase, 3 to 5 meetings were held with the key figure group of each case. In the first meeting it was identified to what extent the country cases met the selection criteria.

Case selection criteria were formalized before the development of the game intervention started. The criteria were related to the setting (local), policymaking approach (cross-sector), target group (youth), willingness to participate and phase of the policy process (working towards an implementation plan). In this study, local level refers to the governmental authorities accountable for local HEPA policies. Depending on each country, the focus was more on local/municipal or regional/county level concerning a specific geographical area with several municipalities.

The cases met all criteria, except for the phase of the policy process. In Romania the case was in the phase of developing the policy plan prior to the implementation plan. Furthermore, themes of the policies differed somewhat across countries. In the Netherlands the policy was a HEPA policy, whereas in Denmark HEPA was part of the health policy and in Romania HEPA was part of the sports policy.

In the meetings also main needs (i.e. problems) were identified. Two needs were raised in all three country cases. The first one was to learn more about the stakeholder network in local HEPA policymaking. The second need was enhancing cross-sector collaboration between the involved stakeholders in the network. These two needs were used for the generic frame of the game, see phase 2 in Fig. 1.

Next to needs, it was also important to familiarize with the specific characteristics of the local HEPA policies in the three country cases, see Table 1. This was also done during the meetings with the key figure group. The research teams improved their understanding of where to focus on in the game, what content should be embedded in the material and who should participate in the game later on, to simulate the real-life policymaking process.

#### Systems analysis of selected cases

For each case, a systems analysis was conducted. The analysis examined the local HEPA policymaking process in terms of key characteristics, i.e. who are involved (the main stakeholders), what is their role and position and how do they relate to each other. The analysis resulted in three schematic models of the stakeholder networks [21].

By identifying these key characteristics, insight into the structure and the processes of the local HEPA policymaking process of each case increased. For example, it became clearer to the researchers what role the stakeholders play in the policy process and where the game can intervene to stimulate interaction and collaboration among stakeholders.

#### Comparison of the systems of the country cases

The comparison of the schematic models of the systems analysis was important to form the generic frame of the game (phase 2), applicable to the three country cases. The different stakeholder networks, by means of the schematic models, were discussed among the three country teams in a workshop in order to obtain a good understanding of each other’s local HEPA policymaking process and to search for commonalities between the three cases. The workshop of this interpretative process was led by a game expert. The comparison focused for example on who is responsible on paper, who takes the responsibility, what are difference in the different phases of the policy process, how structured is this process.

##### Stakeholders

In each of the three country cases, many stakeholders were identified to be involved in the policy process. As this policy game was focusing on HEPA policymaking on local level in regards to collaboration among stakeholders, key stakeholders were identified based on their responsibility and their role in this respect. For example, public authorities with several sectors within the authority entity, schools, knowledge stakeholders, care and welfare organizations or private organizations were identified in each of the cases. However, some of them, for example schools, were positioned on different levels in each of the cases. Also within the local public authorities different stakeholders were identified across the three country cases, such as municipality services.

##### Relations

It appeared that in all systems similar relations were identified, and three types of driving forces were distinguished, i.e. hierarchical relations, informal communication and resource driven relations. However, the distribution of the relations and driving forces differed among stakeholders in the three settings.

Relations within the local public authorities and between the local public authorities and the other local organizations were fairly similar in the Netherlands and Denmark. In Romania, the distribution of relations differed quite a lot, especially the influence on the implementation of the policy. Where in the Netherlands and Denmark the relations seemed to have a more structural basis between organizations, these relations seemed to be more temporary and project-based in Romania. Also the influence of national level on local organizations in regards to the HEPA implementation plan seemed to be more substantial in Romania. Details on stakeholders and their relations are described in Spitters et al. [21].

As a structural nature of relations is an essential precondition for collaboration and the focus of the game was on stimulating collaboration within networks, the relations in the Dutch and Danish case were used as a starting point to develop role descriptions for the generic frame of the game. Furthermore, accountability, as part of the formal relations, was one of the main driving forces. In the Netherlands and Denmark, the local municipality was held accountable for the policy process and was therefore a dominating factor and used to frame the policy game. However, the responsibility of the implementation of the plan differed, from one stakeholder responsible in the Netherlands, to a common good in Denmark. In Romania, the responsibility was not described as explicitly as in the other two cases. These differences in accountability were used in phase 3, tailoring the intervention, to fit the generic frame of the game to each of the three cases.

### Phase 2: Designing the game intervention

The game was conceptualized as a learning experience for supporting collaboration between stakeholders in the policymaking process in three different cases. Before content was given to the generic frame of the game, the research group decided to develop the game as a role-play simulation close to reality, called In2Action. In2Action was a one-day, face-to-face meeting of real-life stakeholders involved in the local HEPA policymaking process. In2Action mimics the policy development process with the intention to let real-life stakeholders identify, experience and act upon the previous identified problems. The specific aim of the game was to facilitate interaction and collaboration between real-life stakeholders, while developing an implementation plan for the local HEPA policy. Participants in the game were collectively addressing the aim of the game over the course of approximately 6 h.

#### Generic frame of the game

To develop a generic frame, several building blocks were formed, in line with Peters et al. [39], which gave content to the game, i.e. the script, main roles, supporting materials, accounting system, the course of the game and the facilitator. The content was based on the previously identified theme, stakeholders and their relations. Aspects less relevant to the cases (noise) were left out of the game to keep it efficient [23, 24, 29, 39]. The content of the building blocks are presented below. Table 3 shows a summary of the generic frame of the policy game intervention In2Action. For a more detailed explanation of the design of the game we refer to the project final report [40].

**Table 3:** Generic frame of the policy game In2Action

The policy game In2Action	
Kind of game	Role play; Real-life network game
Game environment	Simulated municipality (comparable to real-life)
Duration game	One day event of about 6 h
Game facilitator	- One facilitator leads the group of participants. - Plays the role of City Council to approve developed HEPA^a^ implementation plan, based on the intervention cards (ideas) handed in.
Participants	Local and regional/county stakeholders who are or should be involved in the local HEPA^a^ policymaking process in the country case.
Roles in the game	- Each participant has a role close to their actual role/task in real-life. - Nine roles are developed. - Each role is played by a team of 2 to 3 participants. - Additional roles, played by one of the members of the research teams: National Science Academy and City Council.
Starting point	- A HEPA^a^ policy of the simulated city is approved by the City Council - Teams of participants (roles) are asked to work on a HEPA^a^ implementation plan taking into account the objectives of the strategic public health policy
Game theme	To develop a HEPA^a^ implementation plan in collaboration, to achieve the objectives of the approved strategic local HEPA^a^ policy.
Supporting material	- Intervention cards; developed intervention cards, ideas are written down and form the implementation plan and documented collaboration and use of knowledge - Units to support intervention cards - Newspaper, for inspiration of ideas and indicating needs - Map of simulated municipality - Strategic local HEPA^a^ policy - Statistical health report
Course of the game	1. Introduction by facilitator 2. Familiarization with the material by participants 3. 2 cycles 4. Debriefing session

^a^ *HEPA* Health Enhancing Physical Activity

### Building block: The script

The script is the foundation of the game and contains the general description of the simulated safe environment, to learn and experiment. Because of its dominating character in local public health policies, accountability was put central in the game. The common needs across countries were used to create the script and the aim each participants should achieve. Second, interaction and collaboration between stakeholders was put central as main problem to solve. This resulted in the following game theme: to develop in collaboration a cross-sector HEPA implementation plan based on the approved strategic local public health policy of the fictive municipality. Laws and regulations related to public health were expected to be a common good.

### Building block: Main roles

There were two different roles in the game: 1) The played roles by participants, roles in the game which were similar to the function of the participants in real-life; 2) The pseudo roles, which could be taken up by the game leader(s) on request, think of the media or mayor from another municipality.

In search for commonalities across countries, the following nine key categories of stakeholders were identified and created for the purpose of the game: 1. the local authority, for example in the Netherlands the board of mayor and aldermen, and in Romania the city council; 2. and 3. at least two sectors within the local authority; 4. sports organizations, providing knowledge; 5. public health organizations, providing knowledge and health status; 6. education; 7. health and welfare organizations; 8. private organizations and 9. the community/civil society.

#### Role description

To each role, a specific objective was assigned, in accordance to the real-life situation (and based on the information received following discussion with local stakeholders). As the script, the description of roles was kept close to reality. In the role description, tasks were outlined on how to reach the subscribed game objective. Each role had their own color in the game to ease the recognition of the processes in the system (for the participants). Tasks and objectives of a stakeholder in real-life and their relations with other stakeholders in the system provided information for the content of the role description. When stakeholders had similar relations to other main stakeholders in the local HEPA policymaking process, they were grouped together in a role, for example private organizations or health and welfare organizations. In addition, conflicting interests between organizations were taken into account, while developing the role description to stimulate negotiation in the game. For example, one organization would like to focus more on physical activity whereas another organization would like to focus more on nutrition or welfare. In Additional file 1, an example is presented of the translation of a real-life stakeholder (a municipality sector within the local authority) to a role description in the game.

### Building block: Supporting materials

Supporting materials were necessary tools and objects used to play the policy game. These were dependent on the game’s objectives. These materials made achievements in the game concrete and visible. The supporting materials were intervention cards, units, a newspaper, a map of the fictive municipality, a strategic local public health policy and a statistical health report of the fictive municipality.

#### Intervention cards

The intervention cards were the most important tools in In2Action. With the intervention cards participants could achieve the overall aim of the game, developing an implementation plan in collaboration, and their own role objectives. Each of the roles had own intervention cards (similar to a form) where participants could fill in their ideas and plans to provide content of the implementation plan. The cards contained an area to show collaboration with other stakeholders, in ways of shared interest (to be filled in by roles using their stamp) and support (to be filled in with x units) to a developed intervention. Shared interest were achieved by interaction and knowledge exchange. A second area was created to indicate what kind of evidence was used. Together the developed intervention cards formed the local HEPA implementation plan and showed how much stakeholders collaborated with each other in terms of support, interest and knowledge exchange.

#### Units

Each of the roles had a certain amount of units to use for their own or for other’s intervention ideas.

#### Newspaper

The newspaper was developed to help participants start and to give ideas. The newspaper was created with news items of the fictive municipality. Some of these news items addressed also conflicting interest in regards to the objectives of each role. This conflicting interest was embedded in the game to stimulate interaction between participants.

#### Map

The map of the fictive municipality was developed to visualize the municipality for the participants, including items described in the newspaper.

#### Public health policy

The strategic public health policy described the objectives of the fictive city (i.e. in regards to HEPA policy development) and was approved by the city council. The HEPA implementation plan should meet the objectives as stated in this policy.

#### Health report

The statistical health report, containing socio demographic data, was created to stimulate use of evidence in the game. The report was based on realistic data.

Additional tools were used to make the different participants visible in the room (e.g. by using similar colors for materials for each role and nametags). These tools completed the game.

### Building block: Course of the game

**Fig. 2:**
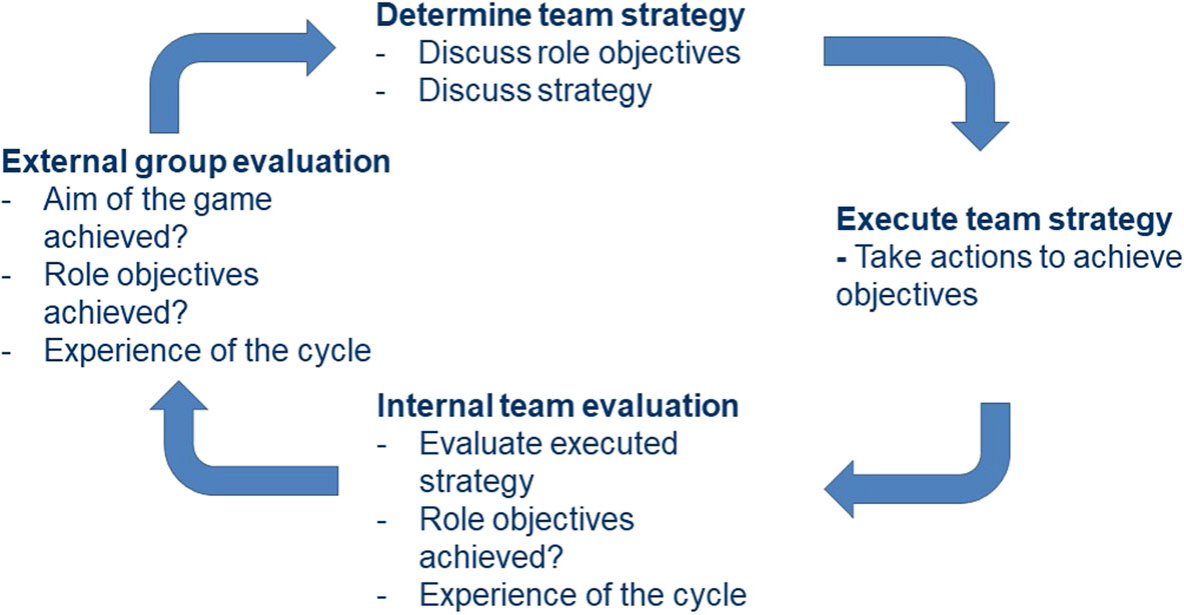
The structure of a policy game cycle

The game started with an introduction by the game facilitator of what participants could expect and to which role they were assigned to. The roles were introduced by the game facilitator at the beginning of the game. The participants were stimulated to interact and negotiate between teams to achieve their own objective and the overall theme of the game. The next step was to familiarize with the roles, their own and the others, and the other materials, i.e. the newspaper, the map, the intervention cards and statistical health report. This was followed by two cycles, each consisting of a strategic internal discussion, for determining team strategy, an external negotiation phase, to execute their team strategy, finalized with an internal team evaluation and a group discussion, the external group evaluation, see Fig. 2. In the group evaluation the learning experiences and achievements (dependent on choices of participants) were explicitly mentioned to take the new insights and experiences to the next cycle. After the two cycles, an overall debriefing session took place to translate learning experiences in the game to useful experiences in daily work and conclude the game.

Each game has rules and thus also In2Action, i.e. the rules of the game and policy rules. The rules of the game had to be followed by the participants and were determined before the game by the game developers and introduced by the game facilitator previous to the first cycle. The policy rules were determined by the participants and came forward during the game. Both types of rules existed next to each other. Furthermore, because time is precious in the game, the facilitator kept track of time.

### Building block: Accounting system

The last building block is the accounting system. This system was related to the expected achievements in the game. Expected achievements were made by the game developers to make the connection between behavior and the achievements produced in the game. In this case, this means whether the overall aim of the game, developing a cross-sector implementation plan, and the objectives of the roles were achieved. Achievements were both subjective, how did participants perceive their achievements and objective measures, the variety of stamps on the intervention cards by means of support and use of knowledge from other stakeholders in the game.

By the end of the internal evaluation of each cycle, the facilitator, playing the simulated role ‘city council’, decided whether the intervention cards, developed by the teams and handed in to the accountable party in the game, would be suitable as an implementation plan to achieve the aims of the strategic local HEPA policy. The acceptance was based on the opinion of the accountable stakeholder in the game, the developed intervention cards, which showed collaboration and support between teams and use of evidence, and the arguments given by the teams afterwards.

### Phase 3: Tailoring the intervention

In phase 3, the generic frame, as described above, was tailored to the local context of the country case, based on the systems analysis of the case and their specific needs, see Table 2.

#### Needs assessment

Next to the common ground for designing the generic frame of the game, specific needs per country case were identified by the key figure group. These specific needs were used to tailor the specifics in the aim of the game, i.e. specifics in the role descriptions and specifics in objectives within the roles. Examples of specific needs were for instance: learn more about the policymaking process in general, learn more about other stakeholders’ roles and interest of other involved organizations, using the game as an initiator for building the organizational network for development of the real-life implementation plan of the country case.

#### Tailoring the generic frame of the game

Before the games could be played in each of the country cases, the generic frame of the game had to be tailored to the specifics of the local case (i.e. stakeholders and their objectives) and its needs (Table 2). Also policy documents and news, available on the internet, were used to tailor the game to the specific case.

For a policy game tailoring means to adapt the content of the materials. Materials that were changed to the specifics of the local case were the artificial environment of the game, the number of roles, the role descriptions and the newspaper. The artificial environment was dependent on the case itself. For example, the map of the city was to some extent similar to the one of the case and news items in the newspaper were related to news and problems encountered by the case itself. In Romania, an additional role was developed next to the nine common roles, to have all stakeholders represented, as identified in the systems analysis. The adjustments in the role objectives were related to the organization(s) the participants were representing and the existing relations between stakeholders in real-life. This included for example adjustments in accountability, because of the differences in the country cases, i.e. local authority or another stakeholder.

#### Implementation of the tailored interventions

The three game interventions were implemented in each country subsequently to each other, starting in the Netherlands in February 2014, followed by Denmark in May 2014 and in Romania in October 2014. The interventions were subject to both quantitative and qualitative assessments at different time points.

#### Evaluation of the game

For the evaluation of the policy game In2Action, a case study design with a mixed methods approach was used, specifically the embedded design, because of the exploratory nature of the intervention and the small groups in each of the cases. Qualitative and quantitative assessments were chosen to analyze the impact of the policy game in the three cases, using a logic framework model, which covered all planned evaluations [40]. The measurements focused on assessing changes in collaboration, organizational network change, leadership and use of knowledge.

#### Ethics

All participants, who took part in the policy game intervention, received written and verbal information on the intervention contents, measurements and use of the data. The ethics procedure met each of the country’s requirements and the REPOPA guidelines.

## Discussion

In this study we have described in detail the development of an innovative game intervention that can help improve collaboration within cross-sector local public health policymaking networks. We took policy games and the gaming literature as a point of departure for this intervention, because games reduce the complexity of reality (i.e. the public health policy process), by focusing on some specific elements of the daily reality (i.e. interaction and relations in stakeholder network) and by removing ‘noise’ (i.e. political agenda, conflicts of interest, other obligations of stakeholders). Hereby games enable us to intervene effectively in the dynamics of the collaboration process to initiate changes [23, 26, 30, 39, 41]. Specifically, stimulating interaction and collaboration between stakeholders in the policymaking process. As Duke framed it: ‘policy games suit very well for circumstances where the objectives are to provide an integrative experience or provide an environment for experimenting with improving group processes’ [24]. Furthermore, the literature indicates that by having the real-life stakeholder network together at one time and going through the consecutive cycles in the game, learning experiences among the game participants may be expected, both in the game as in real-life [41–44]. In all, when all elements of the game are in place learning outcomes in insight in interaction and collaboration in the policymaking processes may be expected.

In the development process of the game In2Action, two challenges were encountered. The first and major challenge was the development of one generic frame for (the three) different EU country cases. Especially making the generic frame of the game applicable and generalizable to case differences in the policymaking processes (e.g. how a local HEPA policy was embedded in each countries’ system) and the diversity in the stakeholder networks were challenging. The intensive interaction with the country research teams were a necessary precondition for the successful development. The second challenge was that good practices and experiences with policy games applied to public health networks of different countries are not represented in the literature. As a result we had to build on generic gaming literature as a basis for this game [23, 24, 29, 39] and looked for guidance by a game expert.

The development of this game consisted of three phases. In the pre intervention phase analyzing the country cases’ policymaking system was a time consuming task [21]. A necessary precondition for this phase was the teams’ knowledge of the country case’s real-life policymaking process, to know stakeholders’ tasks in the process and their relations with others to separate the ‘noise’ from the crucial elements. With this information, stakeholder interaction in the policy process can be stimulated, as strongly recommended by the literature [16–19, 45, 46]. In addition, the game expert was important in guiding the process of finding commonalities across countries, because of some substantial differences across countries.

In phase 2, how to frame the generic game was the major issue because of the substantial difference of the Romanian local policy process, compared with the Netherlands and Denmark. As a result, parts of the game were mainly based on the latter two country cases, with enough ‘openness’ in the design for the Romanian case. Another difficulty in this phase was setting explicit achievements as part of the accounting system. The quality of a policy and its development process is a subjective measure, but explicit achievements in performance of a team and as a group were needed in the game, to make learning outcomes explicit. Therefore, a combination of objective and subjective measures was chosen, stamps on the intervention cards showing the extent of collaboration and plenary group discussions on achievements in interaction, respectively.

In the third phase, the intervention was adjusted to each country case before implementation. Here, the thorough systems analysis was welcomed to translate the generic frame of the game to the specifics of the case [21]. Obviously, because of decisions in phase 2, more adjustments were needed in Romania. Evaluating the policy game intervention will shed light on the effect of the game In2Action in the three country cases [40].

### Strengths and requirements

During the development of the game several strengths of the study were shown. One of the strengths was that the development process of games was followed [23, 24, 29, 39], in close collaboration with key figures of the country cases. The involvement of key figures resulted in a real-life design of the game, with at that time present issues to solve, enabling participants to familiarize themselves with their role in the game and bring up daily life behavior [28, 39].

Another strength is that we developed a generic frame of the game, suitable for three different European country cases. Since the policymaking process varied across the three countries, the game showed to be even more generic and generalizable to other western countries. This is also due to the differences in how HEPA is embedded across the three country cases. In addition, as the Romanian case is similar to other Eastern European countries, the game seems to be generalizable and recommendable for other European countries. Therefore, it is expected that the frame of the game could be applied to many different country cases, as long as the systems analysis is in place and role descriptions and other material are adjusted to the case, accordingly.

In addition to the strengths, a number of specific requirements for developing a policy game should be mentioned. First, the development process of a game, and specifically a game useful for several countries, was time consuming. Each of the countries had to conduct a systems analysis to understand and describe the local HEPA policymaking process in detail, but also had to understand and familiarize with the context of the other two country cases, to find commonalities to develop the generic frame of the game.

This brings us to the second requirement, the need for expertise in developing a policy game. Game expertise is mandatory in the development process, because game development requires a specific background and knowhow. Once the game is developed, application of the game can be done by health promoters or researchers that are familiar with guiding group processes such as role-play.

The last requirement concerns the balance between the generic frame of the game and the tailored game. Usually a game is tailor made from the start. In this study, we developed one generic frame of the game, which was applicable to three country cases. When too many adjustments are necessary, the game might not be suitable anymore and will intervene in the process unintentionally. This requirement was met in this study by developing a game with enough ‘openness’ for the Romanian case. Therefore, although the Romanian case differed substantially from the other two country cases, the Romanian researchers considered that it was still feasible to make the necessary adjustments to have it fit their context. The game will be evaluated to examine how it impacted cross-sector collaboration and evidence integration in public health policymaking in the three country cases.

### Limitations

In addition to the strengths and requirements mentioned above, two specific limitations in relation to the applicability of the policy game to other contexts should be addressed. A First limitation is that the three cases used in this study are not fully representative for all European local and county municipalities. But, as the three cases in this study showed both commonalities and differences in policy systems, the commonalities allowed us to develop the policy game In2Action, leaving enough openness in the generic frame of the game to apply it to all three cases in this study. Thus, also in the Romanian case, where the policymaking process differed most from both The Netherlands and Denmark. The local policymaking process of each of the country cases is supposed to be similar to other cases in that country. Therefore, it can be assumed that these commonalities are also seen in other local public health policymaking processes in other European countries. However, when the policymaking process is very different from the cases included in this study, it may be difficult to implement the policy game in that particular setting.

A second limitation is attached to the necessity to make adaptations to the generic frame of the game, before being able to implement the game in a particular case. Therefore, a good knowledge base of the local public health policymaking process in the country case is required. This asks for good communication between the research team and the local key-figures, being policy officers and advisors. Conducting a systems analysis, the first phase of the development of the policy game comes, is therefore an important and necessary step in making the game applicable for other European cases [21]. Once the systems analysis is executed, cases will understand which stakeholders to involve, how their relations are and what the needs of the case are, to know what adjustments are needed to the generic frame of the game.

## Conclusions

This study introduced and described an innovative way for intervening in stakeholder networks involved in cross-sector public health policymaking, specifically HEPA. We designed and developed a policy game to enhance collaboration between stakeholders active in local public health policy implementation. The focus on collaboration and interaction as essential part of the process, in an artificial setting and reducing, but at the same time resembling closely, the real-life complexity, is the greatest strength of the policy game. Especially because the real-life stakeholder network was brought together in the game, enabling interaction between all involved stakeholders in one day, simulating ‘a pressure cooker’ meeting, able to talk several times repeatedly and in bigger formations than just one-to-one. Second, participants meet new potential stakeholders to collaborate with and work on (existing) relations in the game. In daily work life the stakeholder network will be strengthened including underlying relations, improving knowledge exchange. With some adjustments, the game became tailored and suitable for each of the three country cases. The generic frame of the game is expected to be suitable for other European countries.

## Additional file

Additional file 1: Example of real-life stakeholder to role in the game. On one page it is illustrated how the transmission took place from a real-life stakeholder to a role in the game. This is done by text and by a figure (DOCX 213 kb)

## Abbreviations

HEPA: Health Enhancing Physical Activity
JOGG: Youth on healthy weight
REPOPA: REsearch into POlicy to enhance Physical Activity
