# Supplementary material for: Developing a policy game intervention to enhance collaboration in public health policymaking in three European countries
Source: BMC Public Health. 2017 Dec 19;17:961. doi: 10.1186/s12889-017-4963-7 (PMC5735538; doi:10.1186/s12889-017-4963-7)

**Example of real-life stakeholder to role in the game**

The example describes the transmission of the Dutch real-life stakeholder municipality sector within local authority to a role in the Dutch game, and describes only parts of the role and tasks of this stakeholder.

Several involved sectors in local policymaking can be distinguished in the Netherlands, such as sports and health, both highly involved in HEPA policymaking and urban planning, to a lower extent involved in HEPA policymaking. Municipality sectors play an important role when it comes to the development of policies. Within the sector group a distinction can be made between managers and executive staff. The sectors give guidance to other municipality services and are in direct conversation with other stakeholders outside the local authority, about task divisions and financial decisions. Because of their importance in the local policymaking process, the sectors were given a role in the game. Furthermore, because of the importance of a cross-sectoral approach, the sectors in the municipality were grouped in two types of roles, the ones directly involved in local HEPA policymaking and the ones not directly involved, or at least no direct accountability, in local HEPA policymaking. It is noteworthy that, the relations as identified in the systems analysis were kept in the game. The example is illustrated in the figure below.


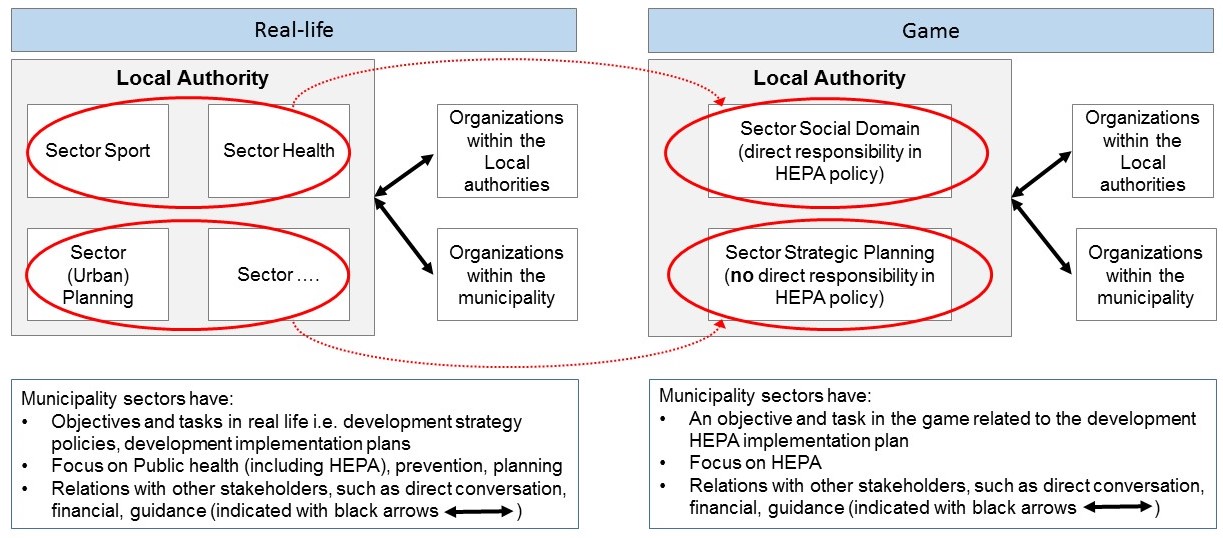

Supplement: Additional file 1: — Example of real-life stakeholder to role in the game. On one page it is illustrated how the transmission took place from a real-life stakeholder to a role in the game. This is done by text and by a figure (DOCX 213 kb) [file 12889_2017_4963_MOESM1_ESM.docx]
